# Resection of Pituitary Tumor with Lateral Extension to the Temporal Fossa: The Toothpaste Extrusion Technique

**DOI:** 10.7759/cureus.5953

**Published:** 2019-10-21

**Authors:** Cordell Baker, Michael Karsy, William T Couldwell

**Affiliations:** 1 Neurosurgery, University of Utah School of Medicine, Salt Lake City, USA; 2 Neurosurgery, University of Utah School of Medicine, Huntsman Cancer Institute, Salt Lake City, USA

**Keywords:** pituitary tumor, sella, transsphenoidal, valsalva maneuver

## Abstract

Transsphenoidal resection of the sellar and suprasellar lesions, whether microscopic or endoscopic, has been traditionally limited by tumors extending laterally to the carotid artery and cavernous sinus. Extended endoscopic or transmaxillary approaches may be warranted depending on these tumor extensions. We describe the use of an intraoperative Valsalva maneuver as a surgical adjunct to the transsphenoidal approach to improve the extent of resection for a favorable outcome. The patient was a 65-year-old woman who underwent resection of a giant pituitary tumor that extended laterally to the cavernous sinus to occupy a volume within the middle fossa. It was the senior author's impression that the lateral cavernous wall was intact at the time of surgery although this is difficult to determine definitively. After a transsphenoidal intrasellar resection of the intrasellar tumor, side-angled endoscopic visualization enabled identification of the breach in the medial cavernous wall where the tumor had invaded the cavernous sinus and ultimately grown into the middle fossa. A Valsalva maneuver was then applied, and the tumor was extruded from the cavernous sinus lateral to the carotid. The significant tumor was removed under direct visualization of the abducens nerve, which was well preserved. Postoperative imaging showed a sufficient extent of resection, and there were no postoperative complications. An intraoperative Valsalva maneuver can be a potentially useful technique for extending tumor resection in cases with a soft tumor and visualization of the opening within the cavernous sinus wall.

## Introduction

Surgical management of sellar and suprasellar lesions, including large pituitary tumors, which have cavernous and middle fossa extension may be problematic. Traditionally, transcranial surgery has been used in pituitary adenomas that are thought to be difficult to resect via the transsphenoidal approach because of retrosellar, retrochiasmatic, subfrontal, or temporal extension; however, the transsphenoidal approach is the preferred method when feasible because there is a higher likelihood of preserving normal pituitary and visual function with less morbidity [1-6].

The authors report a case of a patient who underwent resection of a large pituitary adenoma with tumor encasement of the internal carotid arteries bilaterally and lateral extension to the left middle fossa. An intraoperative Valsalva maneuver was used as a surgical adjunct to the transsphenoidal approach to improve the extent of resection for a favorable outcome.

## Technical report

Patient presentation

**Figure 1:**
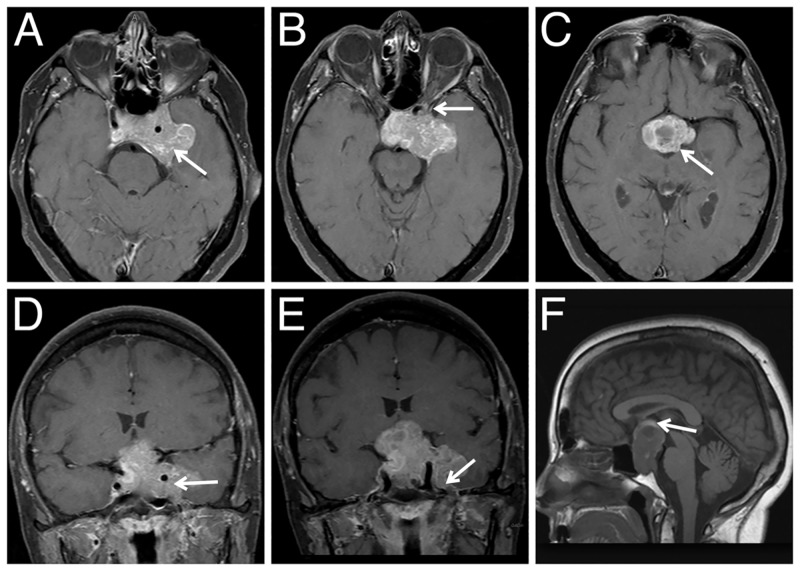
Preoperative contrast-enhanced T1-weighted magnetic resonance imaging (A) Axial image showing pituitary adenoma (arrow) encasing the carotid arteries with posterior extent through the dorsal sella. (B, C) Anteriorly, the tumor extends to but not through the orbital canal (arrow in B), and laterally there is extension beyond the left internal carotid artery with erosion through the left petrous apex (arrow in C). Coronal images showing involvement of the carotid arteries and filling of the left cavernous sinus (D, arrow), with extension into Meckel’s cave (E, arrow). (F) T1-weighted sagittal image showing that the tumor extends superiorly beyond the gyrus rectus and abuts the inferior aspect of the fornix (arrow).

A 65-year-old woman presented to the neurosurgery clinic with the loss of visual acuity for three months and a headache. The patient’s vision was blurry in the left eye, but she could count fingers at a close distance. She was previously seen by her ophthalmologist and underwent cataract surgery in the left eye without improvement in vision. In further work-up, magnetic resonance imaging (MRI) of the brain revealed a large, mixed-intensity pituitary adenoma measuring 5.1 × 2.3 × 4.0 cm (Figure 1). At the time of presentation, she had no evidence of hormonal excess and was taking thyroxine hormone replacement. Pituitary laboratory studies including prolactin, follicle-stimulating hormone, adrenocorticotropic hormone, and cortisol were all within normal limits, indicating that the lesion was likely a nonfunctional tumor, and resection was recommended. After the consultation, the patient agreed to undergo resection of the pituitary tumor.

Surgical treatment

The patient was registered to a preoperative stereotactic head computed tomography (CT) scan using Stealth Navigation (Medtronic, Minneapolis, MN) as previously described [7]. A transsphenoidal approach was used to enter the sella. Endoscopic assistance was used to visualize the intrasellar tumor and identify the carotid arteries bilaterally. The tumor was removed from within the sellar and suprasellar region in the usual fashion. All tumor medial to the cavernous sinus bilaterally was easily removed.

**Figure 2:**
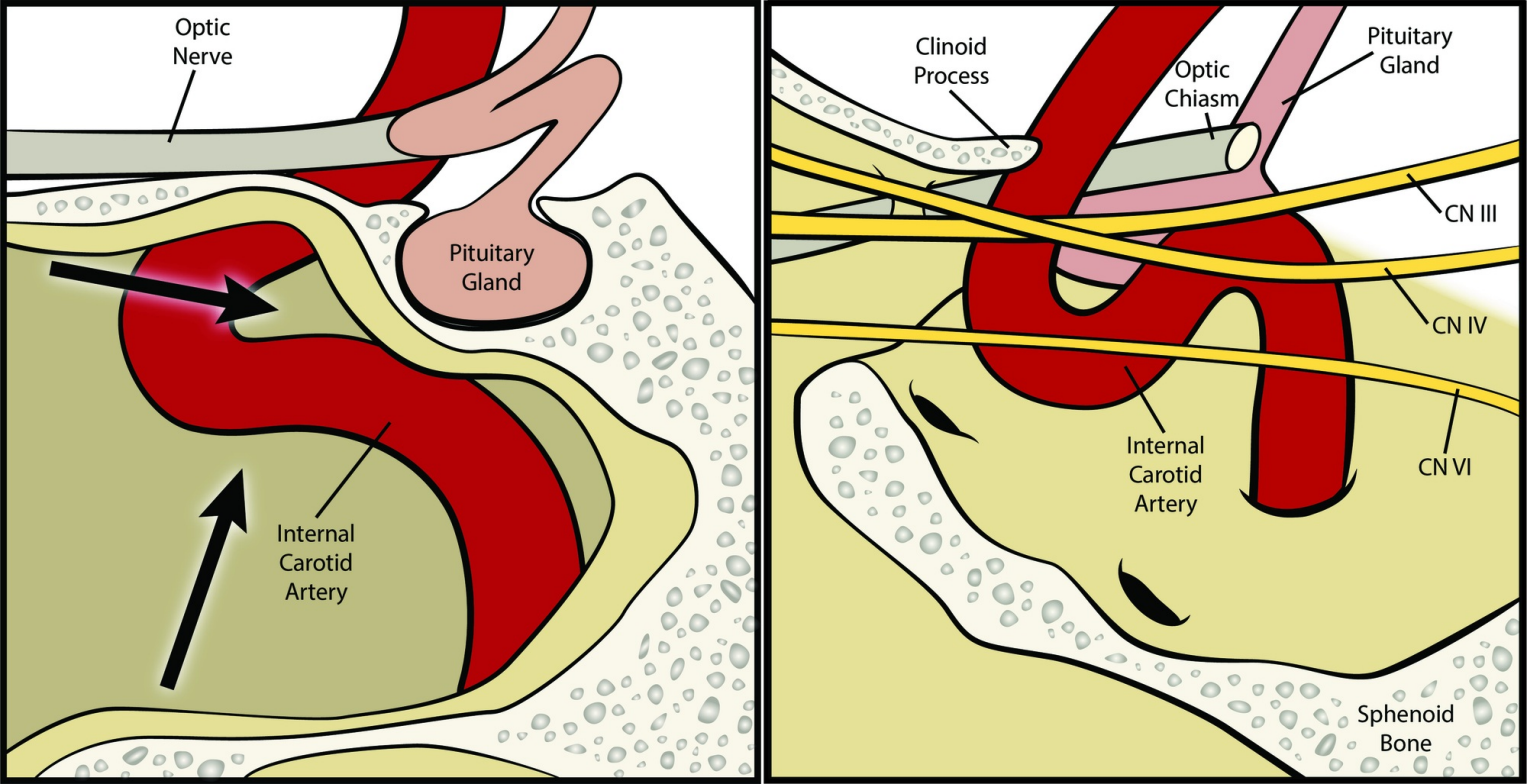
Illustrations in the sagittal plane showing cavernous sinus and corridors for tumor resection (Left) Medial cavernous sinus and corridors where tumor protrusion via a Valsalva technique are shown (arrows). (Right) The position of the cranial nerves within the lateral cavernous sinus is depicted (labeled). Corridors for the Valsalva technique are often widened by tumors growing and extending laterally. © Department of Neurosurgery, University of Utah.

After adequate resection of the intrasellar portion was obtained, attention was turned to the temporal extent of the tumor on the left side. With the assistance of the anesthesiologist, the patient was given a Valsalva maneuver (pressure >30 mmHg, up to 20-second duration, 100% FiO_2_) repeatedly during resection. With the use of a 45° endoscope, the hole in the medial cavernous wall was identified within the anterior loop of the cavernous carotid. Careful dissection in this area enabled visualization of the abducens nerve lateral to the carotid. Because the tumor was soft, the Valsalva maneuver enabled extrusion of the tumor from this corridor, and so it could be aspirated as it extruded (Figure 2). Care was taken not to manipulate the abducens nerve directly, but the tumor extruded both above and below it. The maneuver was repeated until no further tumor was extruded. The closure was then performed as previously described with a fat and fascia graft [8].

Postoperative course

**Figure 3:**
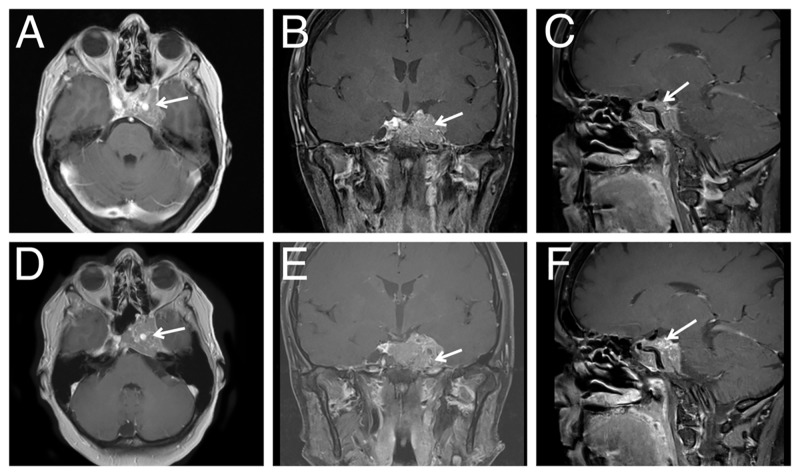
Postoperative T1-weighted contrast-enhanced magnetic resonance imaging (A) Axial, (B) coronal, and (C) sagittal imaging showing decreased tumor burden at one year (arrows). (A) Much of the mass of tumor within the extradural middle fossa space has been removed. There is no longer compression of the left mesial temporal lobe. (A, C) There is residual tumor in the left cavernous sinus surrounding the internal carotid artery. (C) Tumor no longer extends superiorly to the fornix. (D) Axial, E) coronal, and F) sagittal imaging at three years showing similar tumor burden (arrows).

Postoperatively, the patient was monitored in the neurointensive care unit. Pituitary laboratory tests done on postoperative day 2 indicated a cortisol level of 27.2 μg/dL and a prolactin level of 5.7 ng/mL (within normal limits). She was restarted on her previous dosage of levothyroxine. She demonstrated a partial cranial nerve III palsy and was noted to have subtle double vision on upward and downward gaze, both of which improved by three-month follow-up. A mild abducens (lateral rectus) paresis was noted, which improved in the first few weeks postoperatively. The patient’s afferent visual function was normal in both eyes according to ophthalmology records at three-month follow-up. Postoperative pathological evaluation revealed that the lesion was a World Health Organization grade 1 pituitary adenoma. Postoperative MRI showed some expected postoperative residual tumor, which remained stable at one and three years with no further treatment (Figure 3). The patient's scan at three years was concerning for stable to slight interval growth in the tumor; she has been scheduled for stereotactic radiosurgery in the near future.

## Discussion

Various resection strategies for sellar masses include microscopic or endoscopic transsphenoidal techniques as well as open cranial approaches (e.g., frontal, pterional, interhemispheric) [5,9-10]. Decisions about approaches often depend on tumor size, location, and operator experience. Tumors extending lateral to the carotid (e.g., Knosp grade 2 or higher) often present challenges during transsphenoidal approaches, requiring extended endoscopic techniques [11-13]. Similarly, lesions extending lateral and beyond cranial nerves create a challenge endonasally [1-2,14]. The case here suggests a technique to improve resectability via the transsphenoidal technique in a scenario that would typically be treated with either a transcranial approach or a staged transcranial and transsphenoidal approach [15]. Alternatively, an endonasal approach or transmaxillary approach may be used in cases with the tumor extending laterally to the carotid artery, but the manipulation of cavernous cranial nerves will be inevitable.

Surgical treatment of giant pituitary adenomas, formerly referred to as giant pituitary adenomas, is not uncommon. Pituitary adenomas account for 10% to 15% of all intracranial tumors, and of these, it is estimated that 5% are considered “giant” when using a maximum diameter of ≥40 mm [1,16-17]. Resection of large pituitary adenomas is challenging because of the proximity of these tumors to the carotid arteries, the circle of Willis, optic pathway, and oculomotor nerves. When possible, transsphenoidal surgery is preferred because it is associated with lower postoperative morbidity and a higher likelihood of preserving normal pituitary and visual function when compared with transcranial surgery [1,3-4]. Traditionally, tumor extension beyond the sella, specifically lateral extension, has made a transsphenoidal resection alone insufficient for lateral tumor access; however, the use of a Valsalva maneuver as described here can enable resection of the more lateral extension of tumor (Figure 2).

There are a limited number of articles that describe adjunct intraoperative measures to deliver the extrasellar components of large sellar lesions. Many of these maneuvers increase intracranial pressure and force the peripheral tumor to descend into the sella. Reported techniques include jugular vein compression, injection of air via the lumbar drain, and Valsalva maneuvers [14,18-19]. However, the utilization of the Valsalva technique remains limited in the current discussion of extended endonasal and lateral cavernous sinus approaches, whether by microscopic or endoscopic methods. In addition, many of the previous cases tend to focus solely on endoscopic access to the suprasellar component of the tumor without mention of accessing lateral extension or providing specific case examples.

## Conclusions

By providing a case example, this article shows that the use of intraoperative Valsalva maneuver may make transsphenoidal surgery a feasible option in tumors with far lateral extension. We suggest that this maneuver be tried before direct access to the lateral cavernous sinus and middle fossa if the tumor is soft in consistency. The mild third and sixth nerve cranial neuropathies noted resolved quickly, implying minimal disruption of these nerves.
